# Supplementary figures and images for: Disturbance and climatic effects on red spruce community dynamics at its southern continuous range margin
Source: PeerJ. 2014 Mar 6;2:e293. doi: 10.7717/peerj.293 (PMC3961138; doi:10.7717/peerj.293)

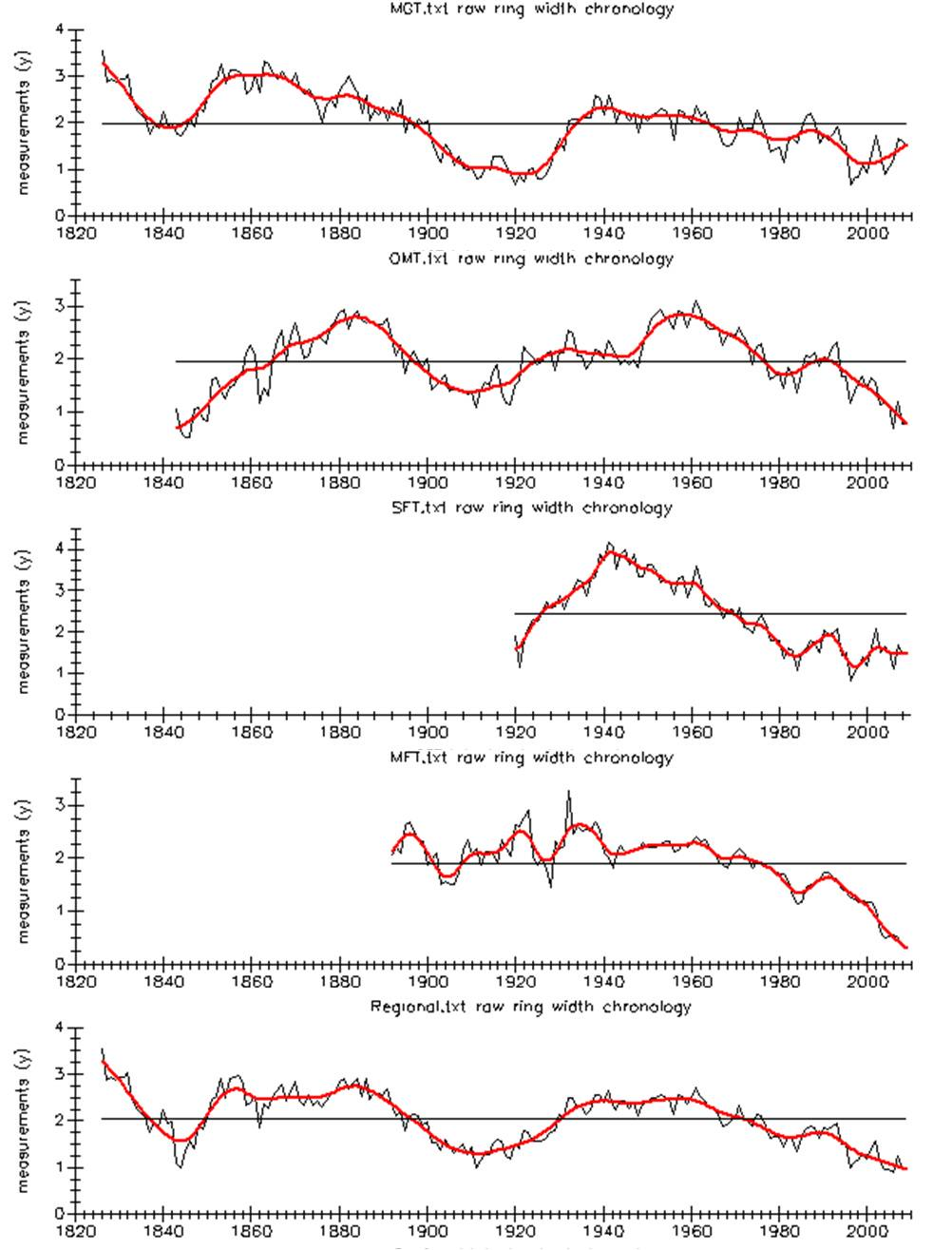

Supplement: Figure S1 — Raw ring width chronologies for each forest and region, showing the general growth patterns before filtering/detrending, with a smoothing spline applied. [file peerj-02-293-s001.png]

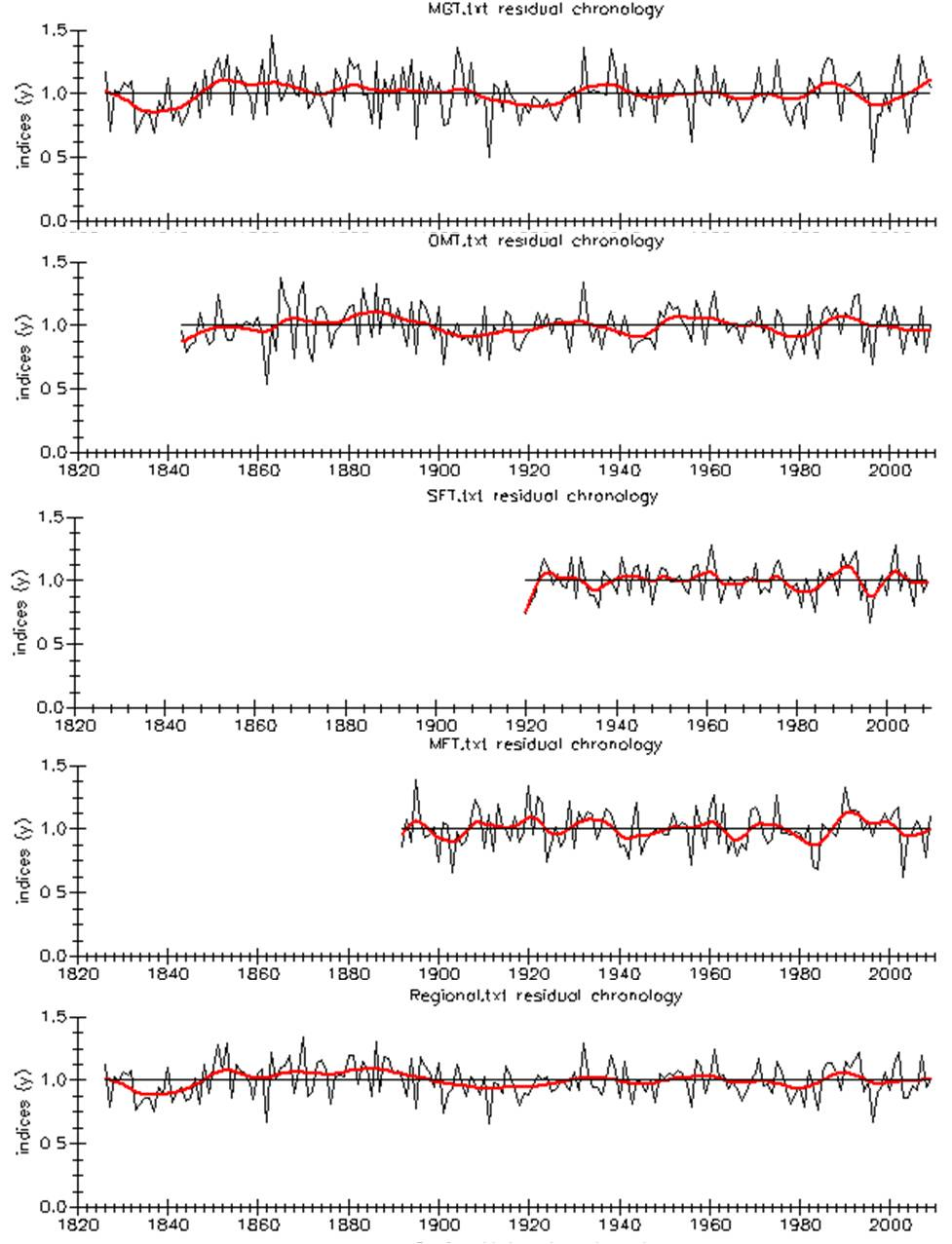

Supplement: Figure S2 — ARSTAN residual chronologies for each forest and region, showing average ring-widths at these sites over time, after detrending, with a smoothing spline applied. [file peerj-02-293-s002.png]

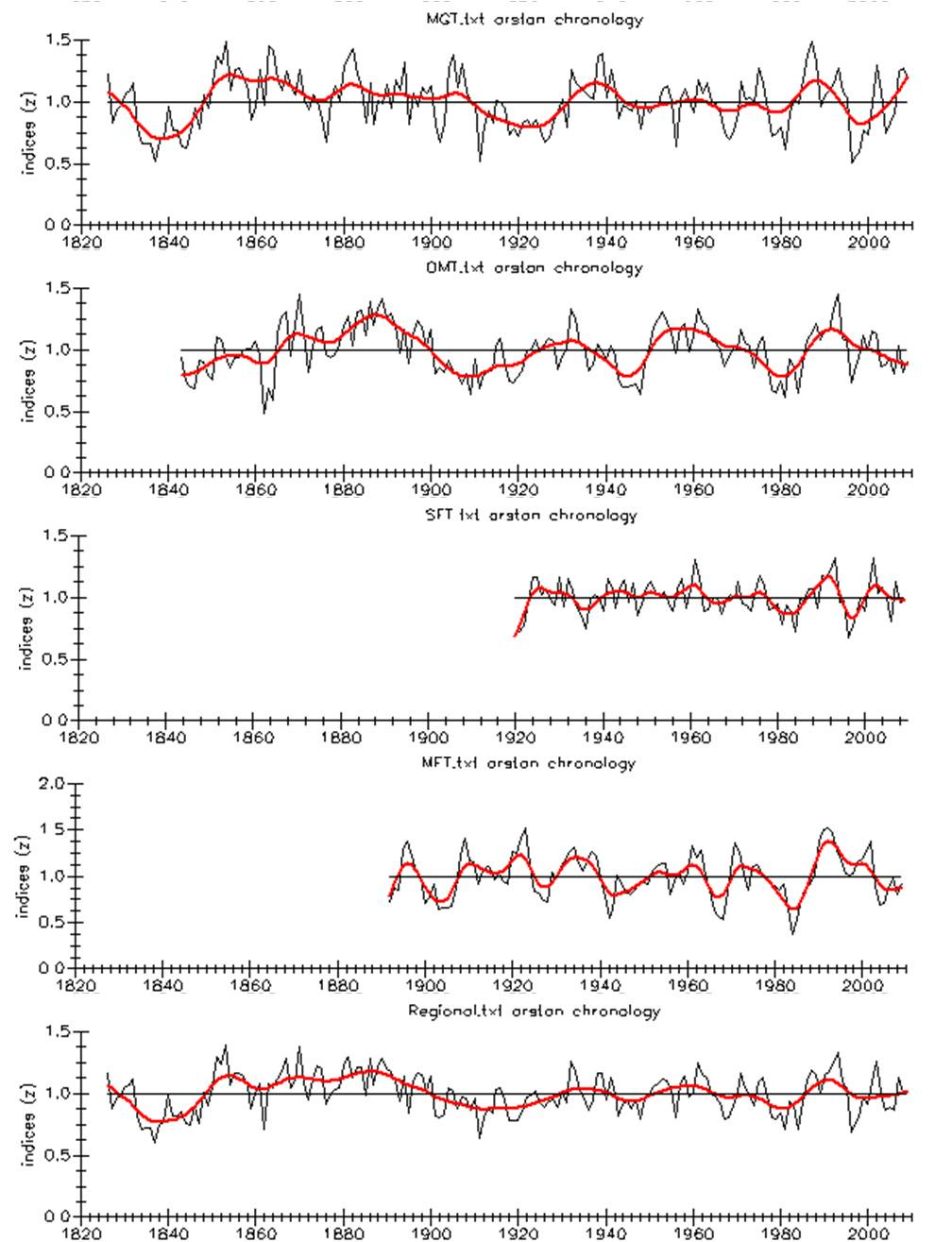

Supplement: Figure S3 — ARSTAN ARSTAN chronology, showing similar trends to residual chronology, but with auto-correlation pooled and added back to the residual chronology, with a smoothing spline applied for each forest and region. [file peerj-02-293-s003.png]

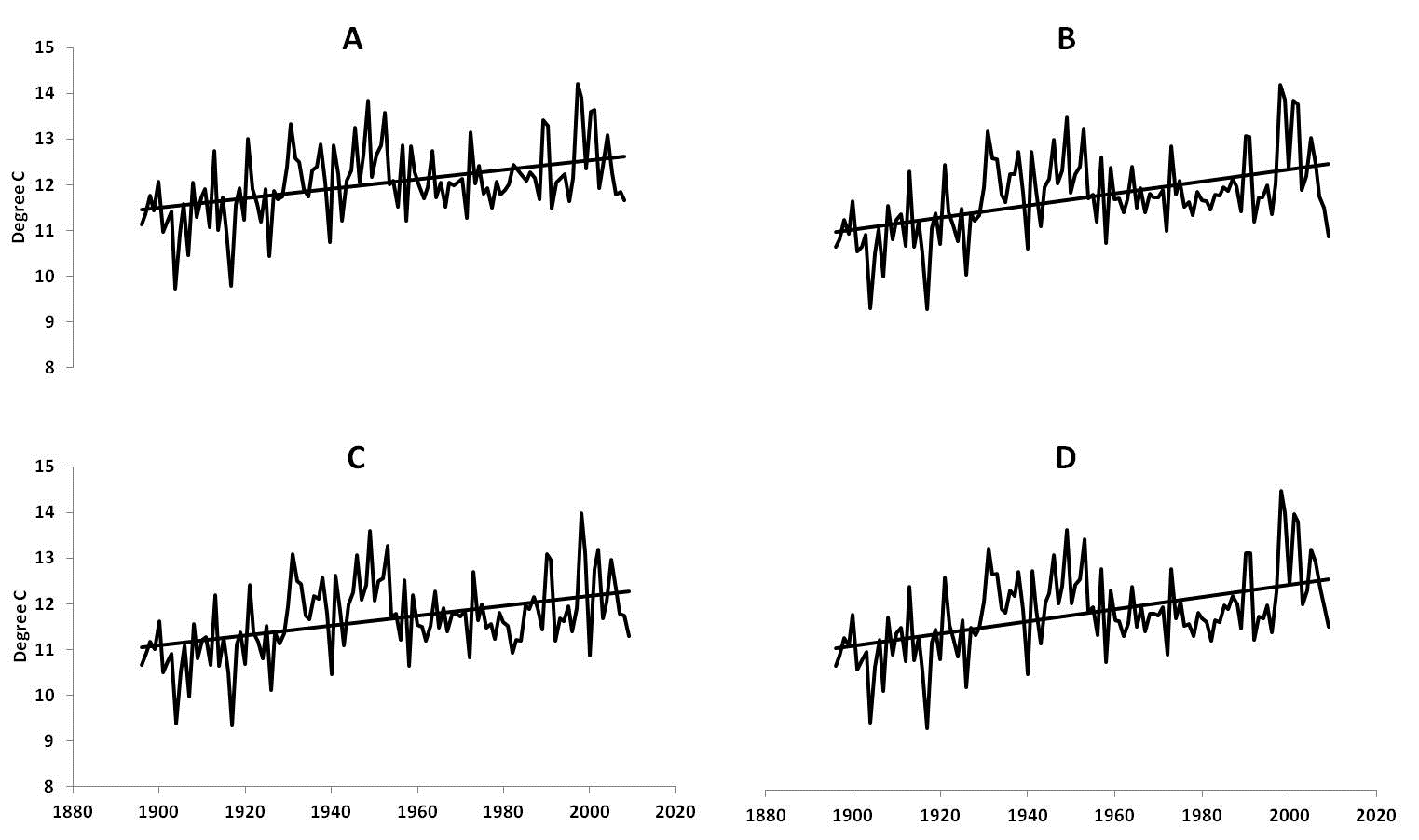

Supplement: Figure S4 — PRISM data for each site MG(A), OM(B), SF(C), and MF(D), showing mean maximum annual temperatures on the left axis, with a trend line showing increasing mean annual temperatures. [file peerj-02-293-s004.png]

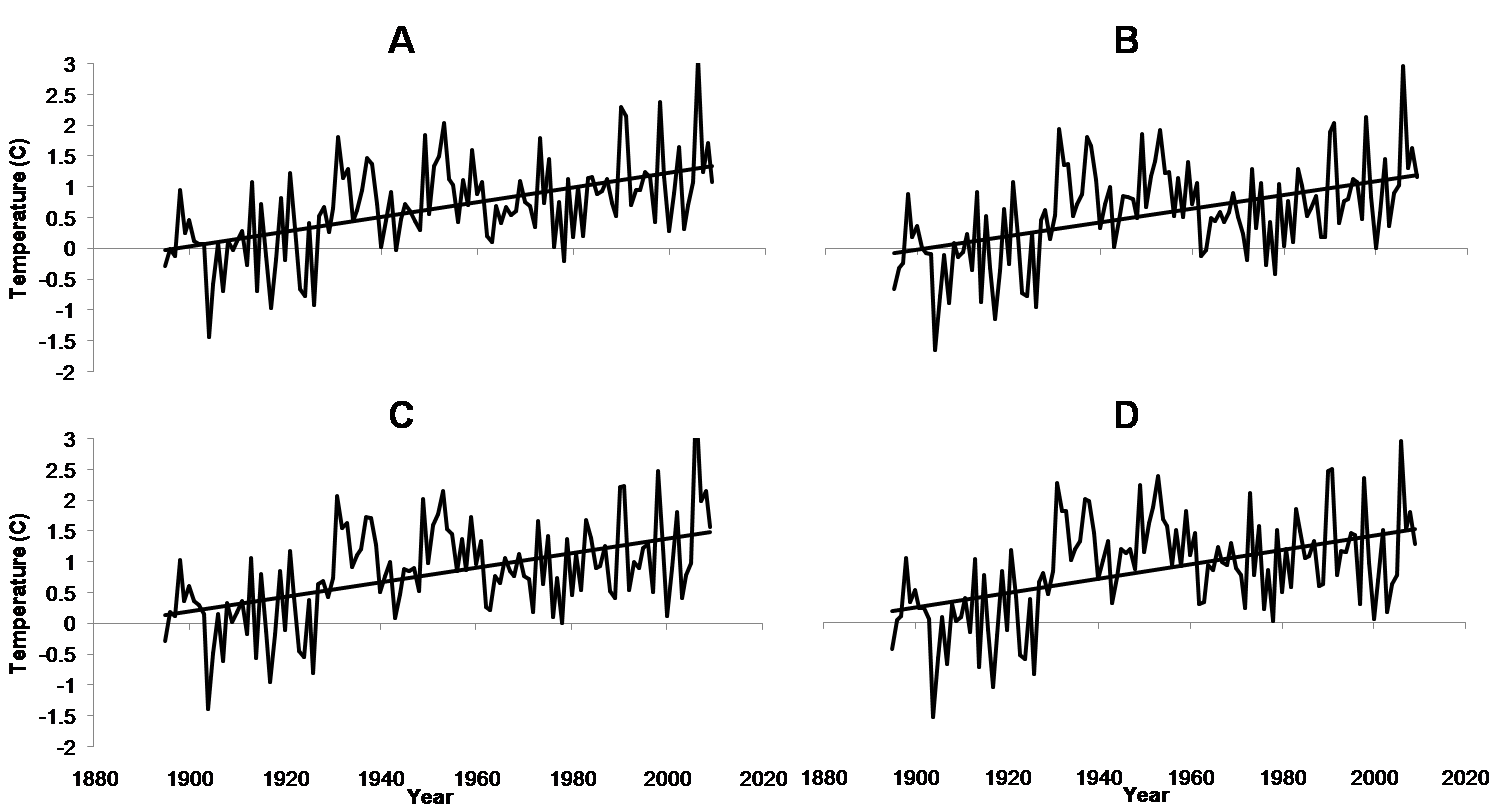

Supplement: Figure S5 — PRISM data for each site MG(A), OM(B), SF(C), and MF(D), showing mean minimum annual temperatures on the left axis, with a trend line showing increasing mean temperatures. [file peerj-02-293-s005.png]

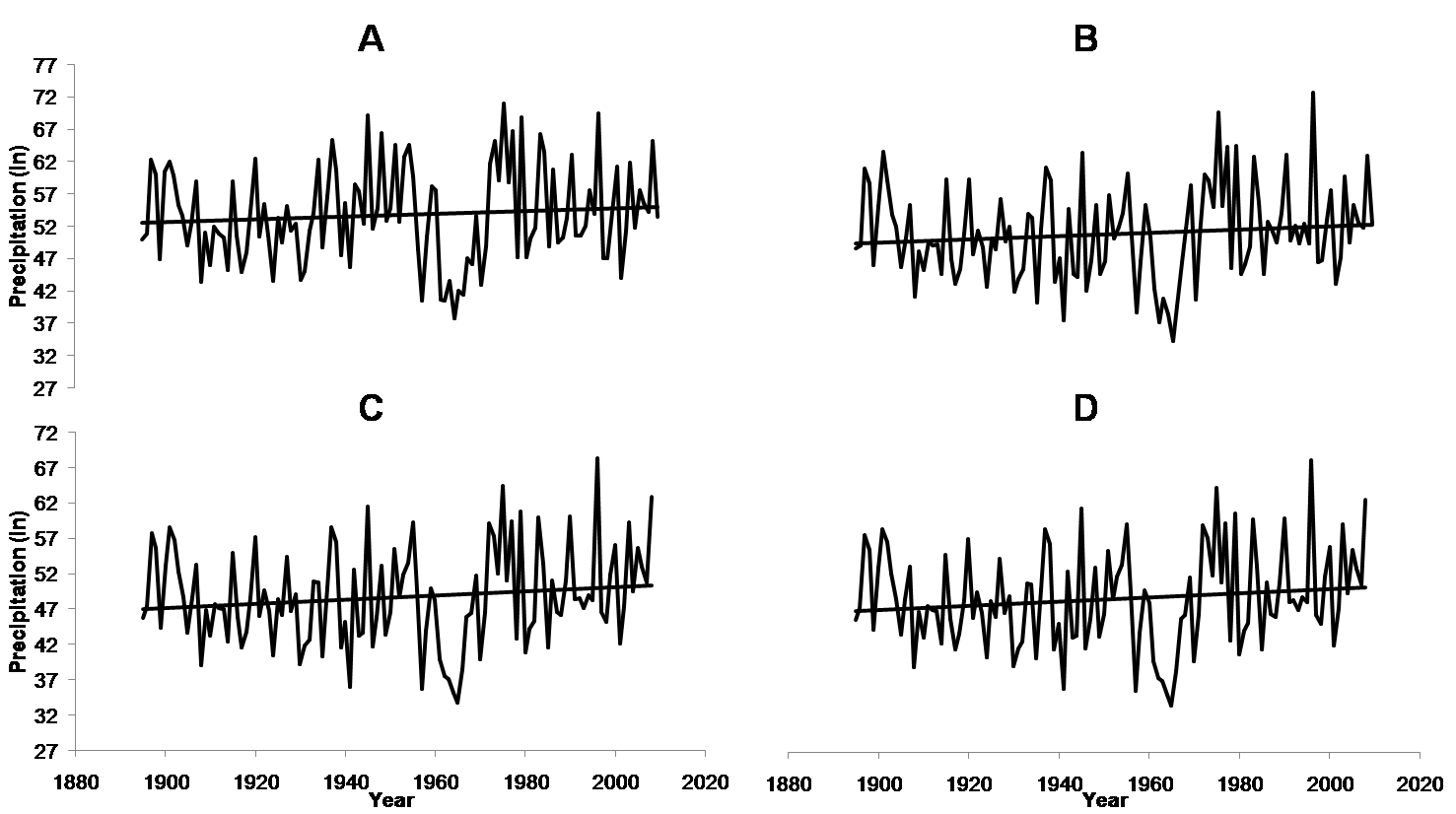

Supplement: Figure S6 — PRISM data for each site, showing mean annual precipitation on the left axis in inches, with a trend line showing minor increases at SF (C) and MF (D). [file peerj-02-293-s006.png]

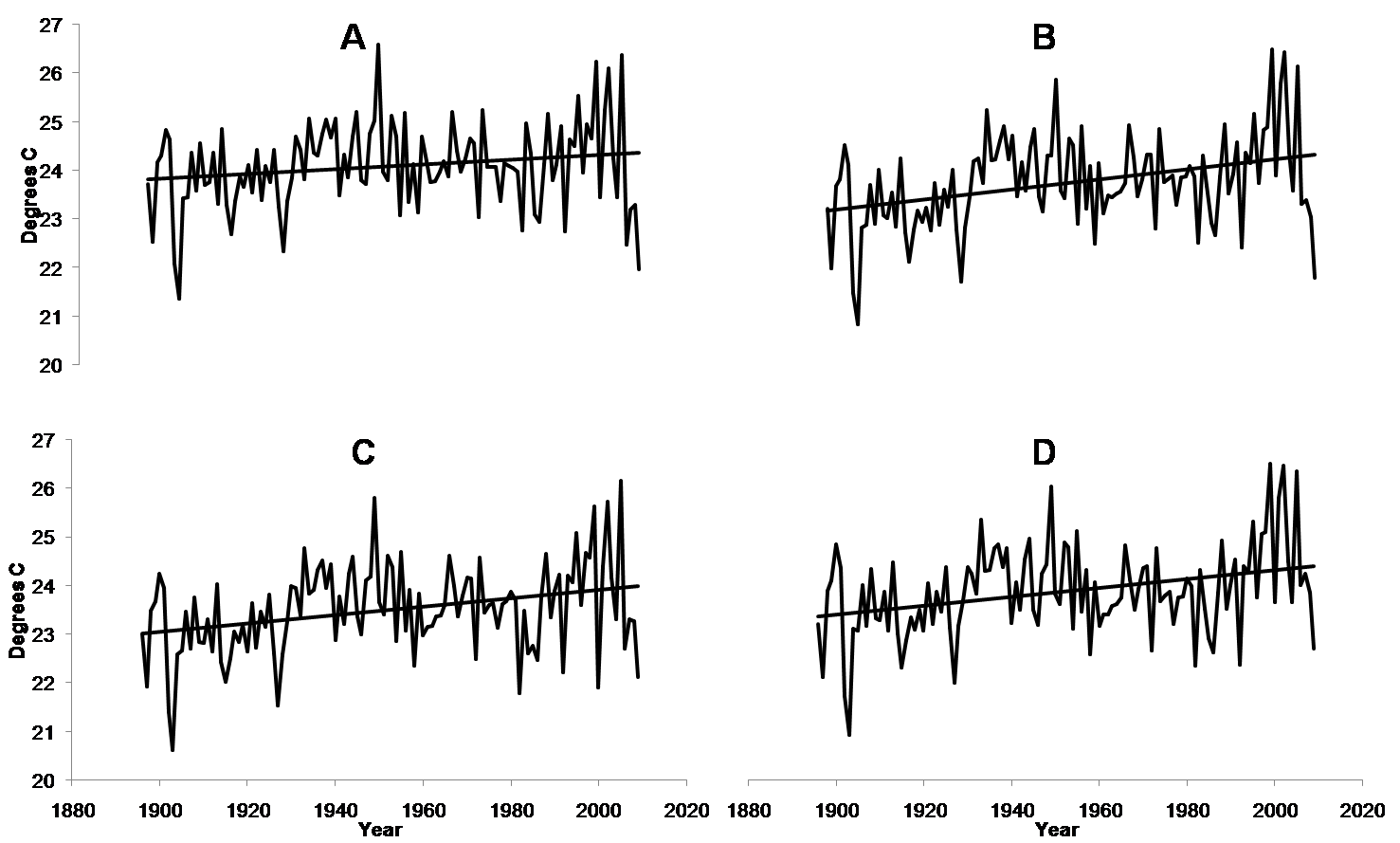

Supplement: Figure S7 — PRISM data for each site, MG(A), OM(B), SF(C), and MF(D), showing maximum temperatures averaged over the summer months of June, July, and August. [file peerj-02-293-s007.png]

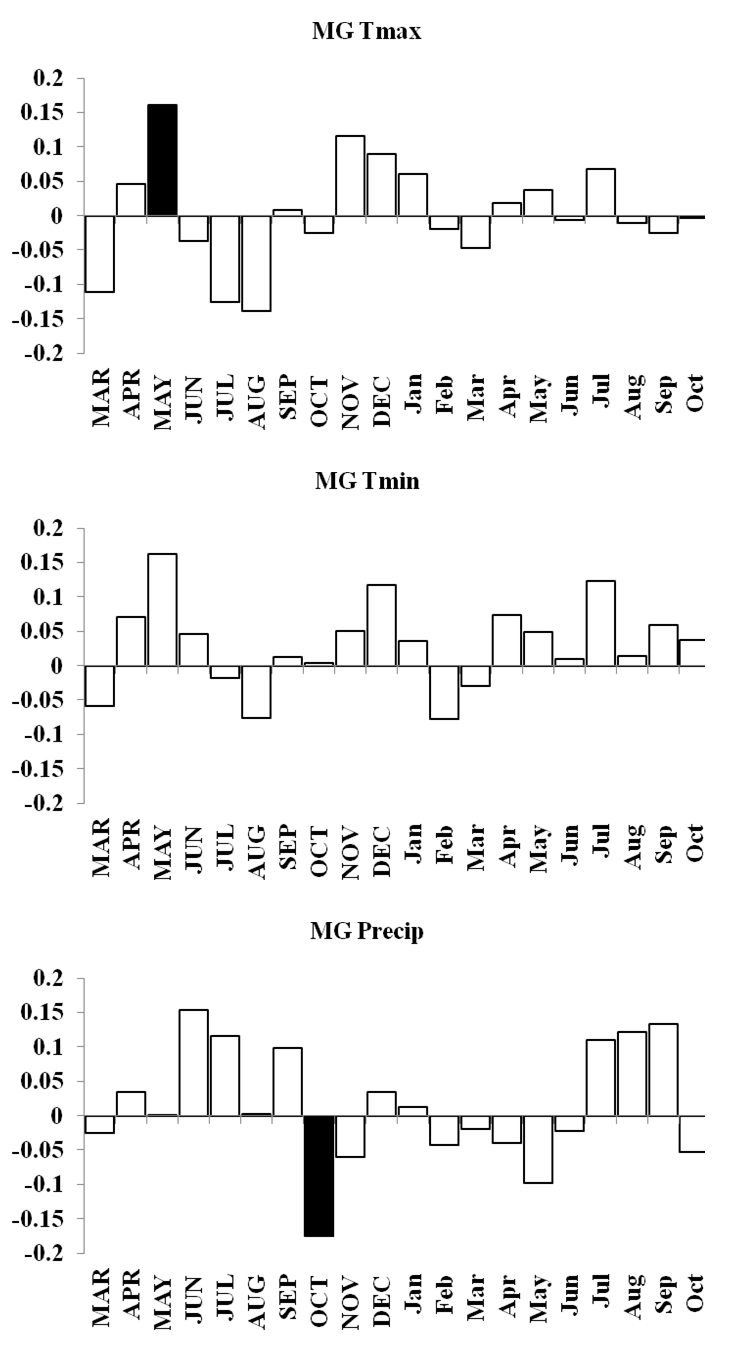

Supplement: Figure S8 — Response function analyses for MG forest showing correlations with maximum and minimum monthly temperatures and precipitation. Black bars indicate a significant relationship. [file peerj-02-293-s008.png]

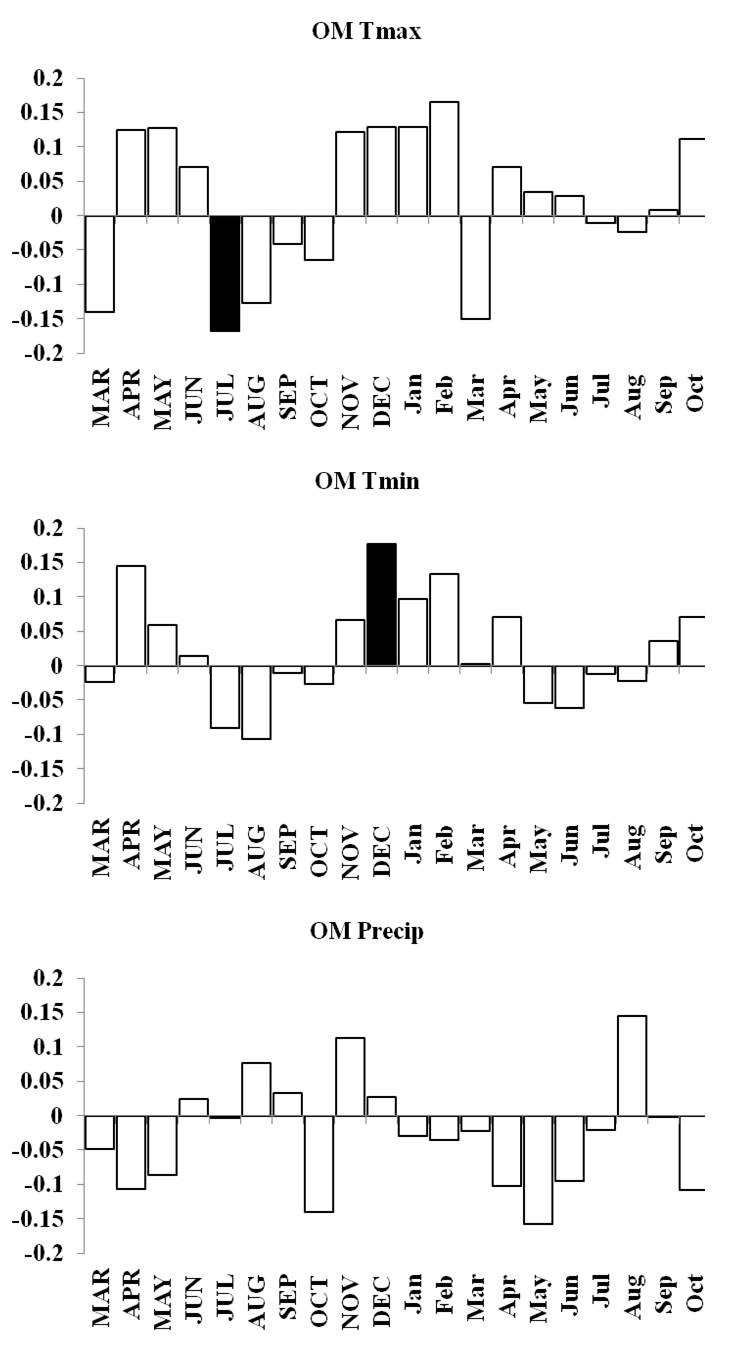

Supplement: Figure S9 — Response function analyses for OM forest showing correlations with maximum and minimum monthly temperatures and precipitation. Black bars indicate a significant relationship. [file peerj-02-293-s009.png]

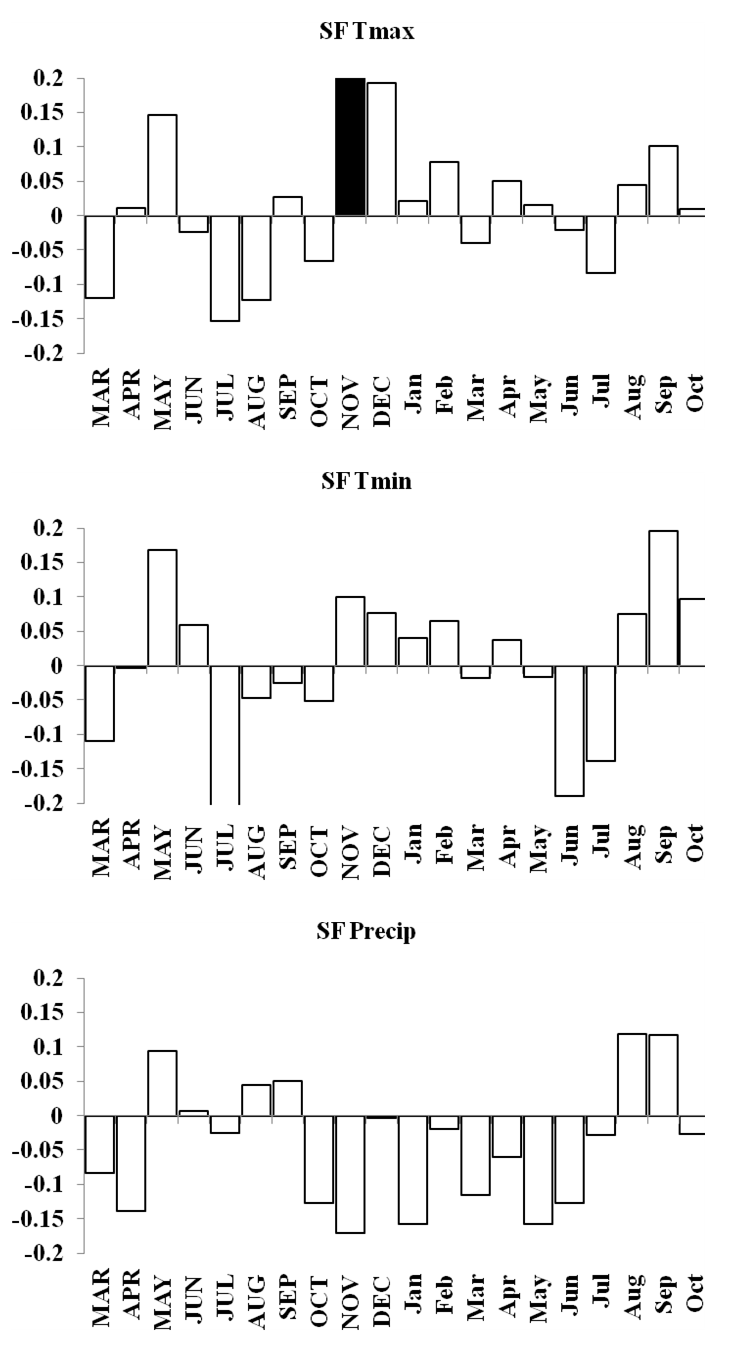

Supplement: Figure S10 — Response function analyses for SF forest showing correlations with maximum and minimum monthly temperatures and precipitation. Black bars indicate a significant relationship. [file peerj-02-293-s010.png]

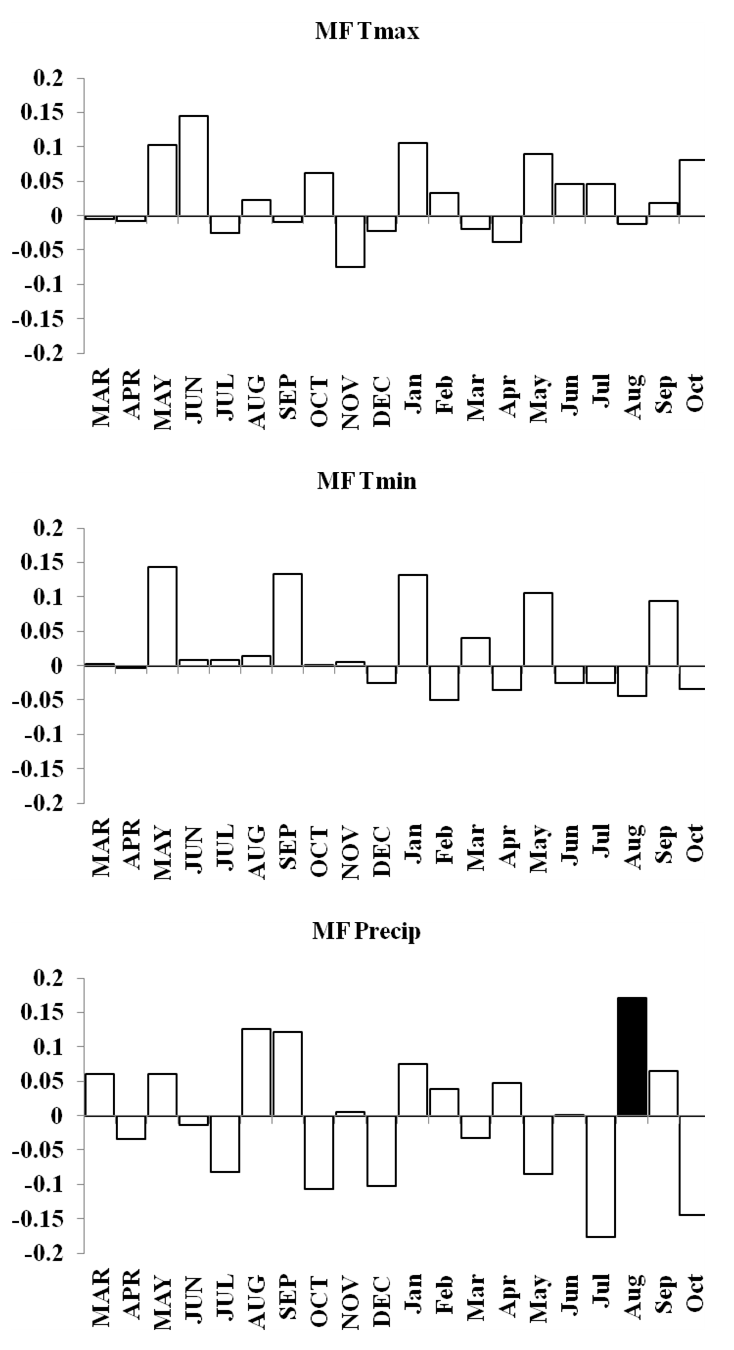

Supplement: Figure S11 — Response function analyses for MF forest showing correlations with maximum and minimum monthly temperatures and precipitation. Black bars indicate a significant relationship. [file peerj-02-293-s011.png]
